# Negative-pressure pulmonary edema after mammoplasty: a case report

**DOI:** 10.11604/pamj.2022.42.15.32010

**Published:** 2022-05-09

**Authors:** Didem Onk, Onur Işık, Faruk Subaşı, Soner Karaali, Ufuk Kuyrukluyıldız

**Affiliations:** 1 Department of Anesthesia and Critical Care, Erzincan Binali Yıldırım University, Erzincan, Turkey,; 2 Department of Plastic and Reconstructive Surgery, Mengucek Gazi Training and Research Hospital, Erzincan, Turkey

**Keywords:** Negative-pressure, pulmonary, edema, mammoplasty, case report

## Abstract

Negative-pressure pulmonary edema (NPPE) is a rare but life-threatening postoperative complication that occurs due to the acute obstruction of the upper airway. In our case report, we present a 25-year-old female patient who underwent elective mammoplasty under general anesthesia and developed NPPE 4 hours after extubation. The patient had a preoperative mallampati score of 3. After routine anesthesia induction, the patient was intubated with an endotracheal tube with a guide wire. Aspiration wasn't observed during extubation. The patient was followed in the post-anesthesia care unit (PACU) for 30 minutes with a saturation of 95% and was subsequently transferred to the service. Four hours after the operation, the patient was re-examined due to dyspnea and shortness of breath. Due to oxygen saturation of 88% and pO_2_of 56mmHg despite mask ventilation, the patient was admitted to the intensive care unit (ICU). A computed tomography (CT) scan revealed extensive diffuse ground-glass opacities and consolidations in both lungs. She did not respond to mask ventilation and was given non-invasive ventilation with continuous positive airway pressure (CPAP). Forced diuresis was induced with furosemide. Tachypnea resolved within 2 hours after CPAP was initiated, the patient did not require oxygen support and COVID-19 polymerase chain reaction (PCR) testing was negative. Subsequently, the patient was discharged to the clinical ward on postoperative day 1. When considering NPPE, early diagnosis and respiratory support are associated with reduced mortality and rapid recovery. Patients who develop laryngospasm during extubation must be closely monitored, and in the case of pulmonary edema, NPPE should be considered in the differential diagnosis.

## Introduction

Negative-pressure pulmonary edema (NPPE) is a rare but life-threatening post-operative complication that occurs due to the acute obstruction of the upper airway [1]. The pathophysiology of NPPE can be summarized as an increase in intrathoracic pressure after difficult inspiration against obstructed upper airways and subsequent fluid translocation to the pulmonary interstitial [2]. In our study, we present a young female patient who underwent elective mammoplasty under general anesthesia and developed NPPE 4 hours after extubation.

## Patient and observation

**Patient information:** a 25-year-old female was scheduled for mammoplasty in the plastic and reconstructive surgery clinic. Her medical history included familial Mediterranean fever, penicillin allergy, and septoplasty 11 months earlier with no anesthesia-related complications. In the preoperative assessment, the patient had a mallampati score of 3. She was pre-operative sent to the internal medicine clinic for consultation, which confirmed that she could undergo the operation without any additional recommendations. She was classified as American Society of Anesthesiologists (ASA) class 2. The patient was taken to the operating room under routine monitoring conditions. For the induction of general anesthesia, she was intravenously administered dormicum at 1mg, propofol at 2 mg/kg, fentanyl at 1 mg/kg, and rocuronium at 0.6 mg/kg. During intubation, she was observed to have a difficult airway, and in addition to direct laryngoscopy, the patient was intubated with an endotracheal tube (internal diameter 7.5 mm) with a guide wire. Anesthesia was maintained with 2% sevoflurane in a mixture of 50% nitrous oxide and 50% oxygen. The operation was completed after 2 hours without any complications. Perioperative blood pressure was 90-105 mmHg, pulse was 85-95 bpm, and blood oxygen saturation was 98-100%. During the operation, the patient was administered 1000 mL of crystalloids. At the end of the operation, she was ventilated with 100% O_2_, and when spontaneous breathing resumed, the residual neuromuscular block was antagonized with neostigmine at 0.03 mg/kg and atropine at 0.01 mg/kg. Retching and agitation were observed during extubation, but no aspiration. After extubation, the patient was admitted to the postoperative anesthesia care unit (PACU) due to a blood oxygen saturation of 92%. She was followed in the PACU for 30 minutes with a mean blood pressure of 80 mmHg, heart rate of 97 bpm, saturation of 95%, and Aldrete score of 10 and was subsequently transferred to the clinical ward without any complaints.

**Clinical findings:** four hours after the operation, the patient was re-examined due to dyspnea and shortness of breath.

**Diagnostic assessment:** she had a respiratory rate of 22 min with rales on chest auscultation. Despite mask ventilation, she had oxygen saturation of 88%, pO_2_of 56.2 mmHg, pCO_2_of 42.6, and lactate of 1.4 and was subsequently admitted to the ICU.

**Figure 1:**
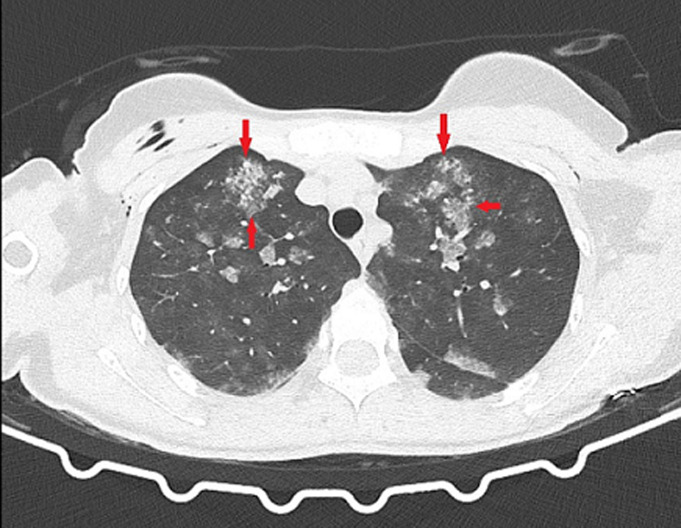
ground-glass opacities and consolidations in the apex part of bilateral lungs

**Figure 2:**
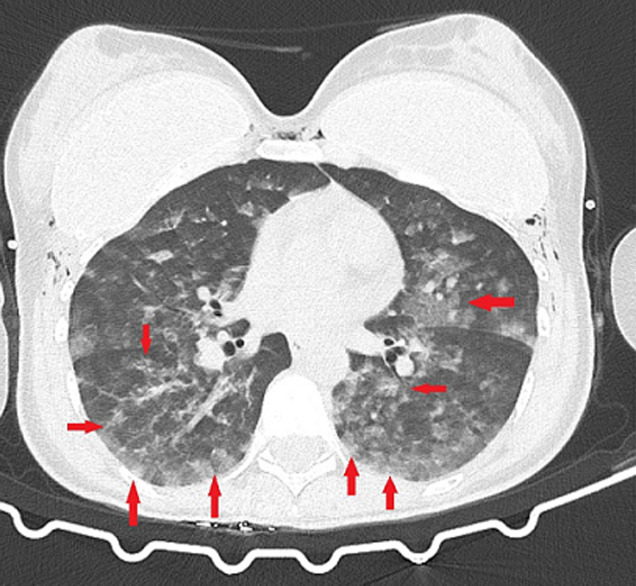
diffuse ground-glass opacities and consolidations in the basal part of bilateral lungs

**Diagnosis:** because of the breast implant, direct chest radiography could not be evaluated optimally and a thoracic computed tomography (CT) scan was performed. The CT scan revealed extensive diffuse ground-glass opacities and consolidations in both lungs (Figure 1, Figure 2). The radiology clinic evaluated the CT findings as “compatible with COVID-19 pneumonia and atypical pneumonia” and recommended clinical correlation. The patient was consulted to the Respiratory Diseases Department, who reported that it was unlikely that she had a COVID-19 infection.

**Therapeutic interventions:** the patient was then ventilated by mask with a flow of 8 L/min. However, blood oxygen saturation did not improve and the patient was given noninvasive ventilation with continuous positive airway pressure (CPAP). A PCR test was performed to rule out COVID-19 infection. Forced diuresis was induced with furosemide and clindamycin was initiated for prophylaxis due to the patient´s penicillin allergy.

**Follow-up:** tachypnea resolved within 2 hours after CPAP was initiated; there was urine output of 1500 mL, pH of 7.34, pCO_2_of 37.6, and pO_2_of 108; and the patient was switched back to mask ventilation. Blood gas values returned to normal, the patient did not require oxygen support, and the COVID-19 PCR test was negative.

**Patient perspective:** the patient was discharged to the clinical ward on posta operative day 1 without any complaints and was satisfied with the result.

**Informed consent:** the patient gave her consent.

## Discussion

Negative-pressure pulmonary edema is a deep inspiratory response that occurs due to the obstruction of the upper airway following laryngospasm in patients under general anesthesia. It is a rare but severe complication that affects 0.05% to 0.1% of intubated patients [3,4]. The increased negative intrathoracic pressure results in an increased venous return to the right heart increased pulmonary venous pressure, and increased hydrostatic pulmonary capillary pressure. The subsequently increased pulmonary capillary wedge pressure results in the transduction of fluid into the pulmonary interstitium. Lung perfusion is impaired due to fluid accumulation, resulting in hypoxia; this, in turn, stimulates the secretion of catecholamines, further increasing pulmonary capillary pressure [5]. Negative-pressure pulmonary edema is more common in young and healthy adults due to strong inspiratory muscles that can produce a higher intrapleural negative pressure. Risk factors include obesity, upper airway obstruction, a short neck, upper airway surgery, obstructive sleep apnea syndrome, and mediastinal mass [6]. Tracheal aspiration, cardiogenic pulmonary edema, intraoperative fluid loading, COVID-19 infection, anaphylaxis, and acute respiratory distress syndrome (ARDS) can be considered in the differential diagnosis [7]. In our case, these diagnoses were excluded because of appropriate intraoperative fluid management, normal echocardiography, no cardiac comorbidities, no predisposing factors for ARDS (such as intraoperative blood transfusion or aspiration), a negative COVID-19 PCR test, and absence of an allergic rash or edema of the uvula. In our patient, respiratory distress during extubation, significant hypoxia and rales on auscultation 4 hours after the operation, and diffuse ground-glass opacities on the CT scan suggested NPPE. The goal of the treatment of NPPE is to correct the upper airway obstruction and the hypoxia [4]. Although CPAP is sufficient in the treatment of most cases, the literature reports rare cases where patients required reintubation [8,9]. Currently, there is no consensus on the use of diuretics as part of treatment and decisions should be made on a case-by-case basis. That said, diuretic treatment is often used in the treatment of NPPE. Early diagnosis and respiratory support are associated with rapid recovery and a good prognosis. Our patient fully recovered within 12-48 hours [10].

## Conclusion

Negative-pressure pulmonary edema is a rare but potentially fatal complication; however, early diagnosis and respiratory support are associated with reduced mortality and morbidity and rapid recovery. If laryngospasm develops during extubation in patients under general anesthesia, they should be followed closely, and in cases of pulmonary edema, NPPE should be considered in the differential diagnosis.
